# Supplementary material for: The relationship between artistic skills and academic engagement among artistically gifted non-specialist university students
Source: PLoS One. 2025 Sep 2;20(9):e0329483. doi: 10.1371/journal.pone.0329483 (PMC12404549; doi:10.1371/journal.pone.0329483)
Supplement: S1 Data — (DOCX) [file pone.0329483.s001.docx]

**The Questionnaire**

**Cognitive** **skills**

| 1 | I have knowledge of the development of art throughout history. |
| --- | --- |
| 2 | I am familiar with artistic concepts. |
| 3 | I have the ability to analyze artworks. |
| 4 | I link the content of artistic works to life and contemporary events in society. |
| 5 | I follow developments in the field of plastic arts. |
| 6 | I have the ability to compare works of art. |

**Performance skills**

| 1 | I have the skill of drawing with a pencil. |
| --- | --- |
| 2 | I have skill in painting using colors. |
| 3 | I make use of appropriate environmental materials to produce my artworks. |
| 4 | I use modern artistic techniques when implementing my artistic works. |
| 5 | I abstain from imitating others while producing artistic works. |
| 6 | I improve my experience by attending workshops and training courses in the arts. |

**Emotional skills**

| 1 | I appreciate artworks in exhibitions. |
| --- | --- |
| 2 | I appreciate works of art from different cultures. |
| 3 | I express my feelings through my art practice. |
| 4 | I can taste the aesthetics of artworks. |
| 5 | I realize the aesthetic dimensions of the colors used in the artwork. |

**The Academic Engagement Scale**

**Academic vigor**

| 1 | I feel active and energetic while studying. |
| --- | --- |
| 2 | I have the desire to study when I wake up in the morning. |
| 3 | I have the ability to study for long hours. |
| 4 | I have great flexibility in my studies. |
| 5 | I persevere in my studies even when things are not going well. |

**Academic dedication**

| 1 | I am proud of my field of study. |
| --- | --- |
| 2 | I feel enthusiastic about my studies. |
| 3 | I achieve my goals by studying. |
| 4 | I try to overcome obstacles during my studies. |

**Academic absorption**

| 1 | Time passes quickly when I’m studying. |
| --- | --- |
| 2 | I feel comfortable while studying my lessons. |
| 3 | I forget everything around me when I’m studying. |
| 4 | I feel happy when I’m studying intensively. |
