# Supplementary material for: The relationship between artistic skills and academic engagement among artistically gifted non-specialist university students
Source: PLoS One. 2025 Sep 2;20(9):e0329483. doi: 10.1371/journal.pone.0329483 (PMC12404549; doi:10.1371/journal.pone.0329483)
Supplement: Attachment — Submitted filename: Response to Reviewers.docx [file pone.0329483.s005.docx]

**Response to academic editor and reviewers**

| **The academic editor** |  |
| --- | --- |
| Ensure that your manuscript meets PLOS ONE's style requirements | Addressed |
| Please note that funding information should not appear in any section or other areas of your manuscript. | Funding information was removed from the manuscript. |
| We note that your Data Availability Statement is currently as follows: All relevant data are within the manuscript and in Supporting Information files.  Please include captions for your Supporting Information files at the end of your manuscript, and update any in-text citations to match accordingly. | Data were provided as supporting information files.  Captions were provided at the end of the manuscript. |
| Review your reference list to ensure that it is complete and correct. | Addressed  Documentation (in-text citations and references) was modified to be in accordance with the journal’s adopted system.  A repeated reference (No. 15) was removed and so numbers of references were changed in the text. |
| **Reviewer 1** |  |
| Check whether all authors cited in the article are in the manuscript’s references, and vice versa. | Checked |
| The article has problems with clarity and coherence in its language, which need to be revised. In the Introduction, it is important to make clear what the objective of the research is, and the reasons that led to the development of this study. In the methodology, it is necessary to make clear some points: - What was the same question asked to the students and to the members of the university’s Gifted Care Unit Council? Was it appropriate to ask the same question? Make it clear which colleges the participating students attended (this was confusing). | The article was checked for clarity and coherence.  The research objective was clarified in the introduction.  The open-ended question is mentioned in the abstract and the study design section: “The questionnaire also had an open-ended question about ways to promote students’ ASs”.  The same question had to be asked for the joint qualitative analysis of both samples’ responses. The researcher wished to identify the suggestions of both the students and the staff members regarding how to enhance students’ talents.  Students’ colleges are mentioned in the participants section. |
| **Reviewer 2** |  |
| The manuscript lacks a more thorough theoretical discussion and definition of “artistically gifted non-specialist students,” especially regarding selection and evaluation criteria. It is recommended to supplement the conceptual framework with references to talent identification systems. Clarify the novelty and theoretical contribution of this study compared to existing literature. | A section was added in the introduction with the heading “Research Context”. A more thorough discussion and definition of artistically gifted non-specialist students is provided in this section. |
| The description of qualitative analysis is too brief. It is advisable to explain the coding process, theme extraction method, and whether triangulation or inter-coder reliability was used. | More details were added in the article.  As to the extraction method, the analysis was conducted by the researcher, given that the task was simple: what are the suggestions offered by students or staff members? It was a matter of how many students or staff members mentioned each suggestion. |
| The regression models show low explanatory power (R² = 0.11–0.13). This should be more thoroughly discussed in terms of potential unmeasured mediators or moderators. The manuscript does not report whether multicollinearity was tested. Including VIF or tolerance values would enhance the robustness of the regression results. | VIF values for the variables were added, which do not show any multicollinearity issues. |
| The description of qualitative analysis is too brief. It is advisable to explain the coding process, theme extraction method, and whether triangulation or inter-coder reliability was used. | The coding process explained. |
