# Supplementary material for: The relationship between artistic skills and academic engagement among artistically gifted non-specialist university students
Source: PLoS One. 2025 Sep 2;20(9):e0329483. doi: 10.1371/journal.pone.0329483 (PMC12404549; doi:10.1371/journal.pone.0329483)
Supplement: Attachment — Submitted filename: Response to editor.docx [file pone.0329483.s006.docx]

Dear Editor,

I would like to inform you that there are no retracted references in the list. Some of them may not appear in Google searches because they are written in Arabic.

Best regards,
Author
